# Effects of the Intervention “Reflective STRENGTH-Giving Dialogues” for Older Adults Living with Long-Term Pain: A Pilot Study

**DOI:** 10.1155/2020/7597524

**Published:** 2020-09-01

**Authors:** Lena Hedén, Mia Berglund, Catharina Gillsjö

**Affiliations:** ^1^Faculty of Caring Science, Work Life and Social Welfare, University of Borås, Borås, Sweden; ^2^School of Health Sciences, University of Skövde, Skövde, Sweden; ^3^College of Nursing, University of Rhode Island, South Kingstown, RI, USA

## Abstract

**Background:**

Long-term musculoskeletal pain is a major, often undertreated, disabling health problem among an increasing number of older adults. Reflective STRENGTH-giving dialogues (STRENGTH) may be a tool to support older adults living with long-term pain. The main aim of this pilot study was to investigate the immediate and longitudinal effect of the intervention STRENGTH on levels of pain, wellbeing, occurrence of depression symptoms, and sense of coherence (SOC) among community-dwelling older adults suffering from musculoskeletal pain compared to a control group.

**Methods:**

The study was semiexperimental with an intervention group and a control group. The effect of a single STRENGTH intervention was reported on the Numeric Rating Scale (NRS) regarding pain and wellbeing. To evaluate the longitudinal effect of STRENGTH, using the Brief Pain Inventory-Short Form (BPI-SF), the Geriatric Depression Scale-20 (GDS-20), SOC-13 at baseline (T1), and six months after the intervention/no intervention (T2), a total of 30 older adults, aged 72 to 97 years (Mdn 86 years), were included consecutively and fulfilled the intervention series (*n* = 18) or untreated controls (*n* = 12).

**Results:**

The intervention with STRENGTH decreases pain (NRS 6 Mdn versus NRS 4 Mdn, *p* < 0.001) and increases wellbeing (NRS 7 Mdn versus NRS 8 Mdn, *p* < 0.001). After a six-month study period with STRENGTH, no longitudinal effect difference was found compared to baseline. Compared to the control group, there was an increasing trend between decreased pain level and increased SOC level for STRENGTH intervention.

**Conclusions:**

This pilot study supports STRENGTH's effect as a pain-alleviating model that provides a decrease in pain levels and an increase of wellbeing in older adults with long-term pain. STRENGTH dialogues could be a useful intervention to provide individually holistic care in older adults living with long-term pain.

## 1. Introduction

Increasing age is often associated with complex health problems, and one predominant and pervasive health problem that tends to increase with age is musculoskeletal pain [1–4]. Long-term musculoskeletal pain is recognized as the main cause of loss of physical and psychological functions resulting in impairments and disabilities with a significant impact on the quality of life (QOL). The loss of functions also limits social participation and independence [3, 5–11]. Furthermore, there is a clear association between this type of pain and the development of depression [9, 12–14]. Research shows that the severity of long-term pain is associated with reduced QOL and increased use of resources, resulting in increased health care costs in society [10]. Regardless of the frequency of musculoskeletal pain and its impact on older adults' lives and society at large, researchers continue to report that this type of pain, like long-term pain in general, is frequently unrecognized, underreported, and inadequately treated among older adults [3, 15–18].

The overall prevailing focus in the provision of care to older adults is the transition of care according to the principle of remaining at home with support from home health care providers. This orientation aligns with the desire of many older adults to remain at home as long as possible [19–27]. However, musculoskeletal pain is recognized as a threat and a hindrance in the strive to remain at home, sometimes with help from home care services, and live a healthy active life as independently as possible. This type of pain is problematic for both the individual and society at large which addresses the need to acknowledge this health problem from a holistic perspective in the planning and provision of care [3, 19–26, 28]. This relocation of care is a crucial challenge for society at large in the provision of services and health care that must focus on ways to support successful aging [16, 29]. This calls for interventions with the potential to decrease suffering and pain and at the same time enhance health and wellbeing while aging in place [30]. However, it is a challenge to tailor individual holistic care that meets the older adults' needs at home and it requires interventions that are individually and holistically tailored in order to preserve and improve health, wellbeing, QOL, and independence [21, 24, 31–33].

Previous research, primarily in institutional settings, has shown that provision of care from multidisciplinary teams, physical exercise, and cognitive behavioral therapy can help patients to cope better with chronic illness and improve self-management of their pain [29, 34–37]. There is growing evidence that these types of intervention can be useful for older adults suffering from pain [38, 39]. However, interventions are needed that embrace living with pain as a whole and not the pain per se. Research shows that daily living with pain is less in pain management than in how to endure the pain and deal with daily life. There is a need for guidance and support in this act of enduring since older adults felt that they are forced into learning to live with pain on their own. They coached themselves throughout daily life using trial and error [32, 33].

The interventions need to promote participation by the users [21] and be designed as an appropriate tool to secure and enhance the quality of person-centered care in a given context [40]. The reflective STRENGTH-giving dialogue (STRENGTH) was developed to address these needs [41]; for an overview of dimensions, see Table 1. It is developed in the discipline of nursing based on knowledge from the dissertations of Gillsjö [42] and Berglund [43]. The theoretical framework for the method STRENGTH is the life-world perspective [44–46], which emphasizes the human experience as the basis for caring and learning, as described by Gillsjö and Berglund [41]. The dialogue in STRENGTH has its basis in the human being's subjective experience of health problems such as pain, existential anxiety, and mental and physical illness in daily living. In caring science, the subjective experience of health entails the sense of wellbeing and the ability to carry out small and large projects that are important in life. A sense of wellbeing includes the ability to balance rhythm, pleasure, courage, meaning, and strength in life [47–49]. The method has the orientation of both a method and an overall approach used to holistically and individually guide and support older adults in finding ways to live a meaningful life despite pain and to fulfill their desire to remain at home as long as possible. The dialogues are carried out by health care professionals like occupational therapists, physiotherapists, and nurses; all have professions using methods grounded in caring science. This method can give the professionals an interprofessional core in the provision of health care [41]. Both the older adult as a recipient of the dialogue and the health care professional as a conveyer responsible for carrying out the dialogues are active in the intervention. This external pilot study is a part of a larger project; results from a qualitatively analyzed study were published earlier [50]. This study adds a quantitative perspective to STRENGTH and is the first quantitative pilot study to investigate the effect of the method on older adults living with long-term musculoskeletal pain.

The main aim of this pilot study was to investigate the immediate and longitudinal effect of the intervention STRENGTH on the levels of pain, wellbeing, occurrence of depression symptoms, and sense of coherence (SOC) among community-dwelling older adults suffering from musculoskeletal pain compared to a control group. A further aim was to perform a basis for a power calculation for a larger study.

The following research questions were posed:

1. Is there an immediate effect of reported level of pain before and after receiving STRENGTH-giving dialogues?
2. Is there an immediate effect of reported level of wellbeing before and after receiving STRENGTH-giving dialogues?
3. Are there longitudinal effects on pain between baseline and after intervention with continuous STRENGTH-giving dialogues compared to a control group?
4. Are there longitudinal effects in reported depression/mental health between baseline and continuous STRENGTH-giving dialogues compared to a control group?

## 2. Materials and Methods

**Figure 1:**
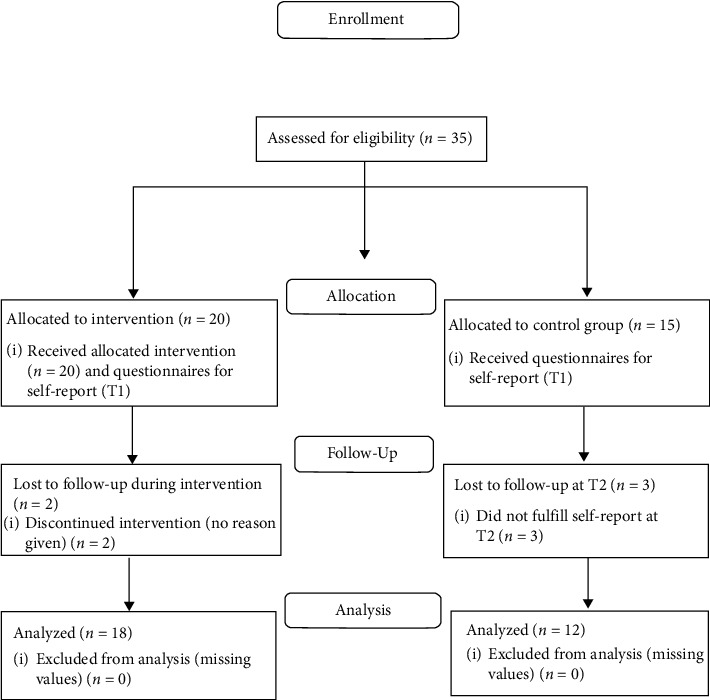
Flow diagram of the participants in the intervention and control groups.

Health care professionals in community-based home health care services (nurses, physiotherapists, and occupational therapists) identified older adults that met the inclusion criteria community-dwelling older adults at age 65 or above who for at least six months have lived with long-term (persistent or regularly recurring) musculoskeletal pain at home and received community-based care services at home. The pain was self-reported and there were no requirements regarding the level of pain, location, or interference of pain in daily living. Twenty older adults (aged 72 to 97 years of age) were included consecutively between January and June 2014; an untreated control group of 15 participants was recruited with the same inclusion criteria (see Figure 1). In terms of cognition, for participation in the intervention and control groups, it was required to understand and answer questions in Swedish and be willing to participate in the intervention or control group. Figure 1 also illustrates the flow of participants throughout the study, as recommended by the CONSORT statement [51].  Table 2 provides background characteristics of the study groups. Finally, a total of 30 older adults (intervention group *n* = 18 and control group *n* = 12), aged 72 to 97 years (mean 84 years), were included consecutively.

### 2.1. Setting

Data were collected in the context of home health care in four communities in the countryside of the western region of Sweden. The participants in the intervention group were recruited from three communities. The control group was recruited from a community nearby that was not included in the STRENGTH intervention. The choice of separate communities for recruitment of participants to the intervention and control groups was made based on the need to avoid spill of effect from the health care professionals in the intervention to participants in the control group.

### 2.2. Procedure

The study was conducted over six months among community-dwelling older adults living with long-term musculoskeletal pain at home. The STRENGTH intervention contained an initial education, followed by a period of four months in which ten health care professionals (seven registered nurses, two physiotherapists, and one occupational therapist) conducted reflective STRENGTH-giving dialogues. Each health care professional carried out dialogues once a week with one or two older adults receiving home health care. The health care professionals had at least three years' experience of providing health care to the population described above and completed a three-day educational program regarding the method STRENGTH intervention. The education was oriented towards physical, psychological, and existential issues with a focus on promoting and preserving health, wellbeing, joy, meaning, and strength in life. To support dialogue and reflection during the STRENGTH intervention, materials such as pictures and booklets were used. The STRENGTH method is further described in research by Gillsjö et al. [41, 50]. In addition, continuous supervision from the second and third authors was provided to support the health care professionals during the intervention period. The choice of time and frequency of the intervention and measure points was based on earlier experiences from research and specialist nurse work experience in the area of gerontology and geriatrics [42].

Pain and wellbeing were reported on a Numeric Rating Scale (NRS) ranging from 0 to 10 by the older adults before and after every STRENGTH-giving dialogue. Also, to evaluate the longitudinal effect, the STRENGTH intervention questionnaires were used to collect baseline data (T1). This was done by the researcher at the time of the qualitative interview. The researcher could further explain the questions and help to respond when needed. Some of the participants had impairments that prevented them from reading and filling in the questionnaires themselves. Since questionnaires could be difficult for older adults to complete, the questions in this study were performed as a structured interview when needed. This was done by the researcher (CG, MB). The researcher could further explain the questions and help to respond when needed. The STRENGTH-giving dialogues and the collection of data before and after intervention were carried out in the participants' homes after acceptance of participation and scheduling appointments. Data were collected before, during, and after the intervention, from January to June 2014. To evaluate the intraindividual experiences of STRENGTH-giving dialogues over time, a longitudinal design was set with a baseline (T1) and a six-month follow-up (T2). The control group only receiving usual care, they already had, answered the questionnaires at baseline (T1) and after six months (T2).

### 2.3. Instruments/Questionnaires

The NRS 0–10 (where 0 corresponds to “no pain” and 10 corresponds to “worst imaginable pain”) ranges from 0 to 10. The NRS was used at the beginning and end of each dialogue to evaluate the level of pain and sense of wellbeing. For wellbeing, 0 corresponds to the “lowest level of wellbeing” and 10 to the “highest level of wellbeing.”

The Brief Pain Inventory-Short Form (BPI-SF) was used to assess the older adults' pain intensity (mean of worst, least, average, and now pain level) of and pain influence (interference) on their daily lives (mean of general activity, walking, work, mood, enjoyment of life, relations with others, and sleep). The questionnaire contains nine items on a scale of 0 to 10 [52]. The Geriatric Depression Scale 20 (GDS-20) was developed as a screening instrument in a clinical setting to facilitate the assessment of depression in older adults [53]. The most common version used is the GDS 15-item short form [53], extended with five items related to sleeping habits, anxiety, pain, and worries about illness in daily living [54]. The GDS-20 is a self-rating scale, but the developers recommend that the test be administered orally by an interviewer, based on the belief that cognitive problems can affect the accuracy of self-reported problems. The GDS-20 has a dichotomous yes/no response choice for each item, with a timeframe of feelings over the past week. A score of 5 or below indicates that depression is unlikely, and a score of 6 or above indicates that the possibility of depression should be evaluated. Scores between 5 and 9 indicate mild depression, while a score of 10 or more indicates moderate to severe depression [54]. The GDS-20 has been found to have high sensitivity and specificity in diagnosing depression [55].

The Sense of Coherence-13 (SOC-13) assessment measures the individual's overall ability to manage difficult situations (coping strategies) and distress [56]. The SOC short version scale consists of 13 items on a 7-point Likert-scale, ranging from “very often” to “very seldom or never.” The SOC-13 scale evaluates perceived comprehensibility (5 items), manageability (4 items), and meaningfulness (4 items). The minimum number of points that can be assigned to anyquestion is 13; the maximum number is 91. A higher score represents a stronger SOC. SOC-13 has been tested for validity and reliability in a number of studies. All instrument versions used were earlier translated and adapted into Swedish.

### 2.4. Statistical Analyses

Descriptive statistics were used for a presentation of background data, and the chi-square test was used for a comparison between the intervention group and the control group. The primary outcome variable is the level of pain. To answer research questions 1 and 2, data were analyzed with the Wilcoxon Matched-Pairs Test; to answer research questions 3 and 4, data were analyzed with repeated-measures mixed ANOVA to evaluate STRENGTH in older adults' reported levels of BPI pain severity and pain interference, depression, and SOC over time and considering potential interactions compared to the control group. The repeated-measures mixed ANOVA met the criteria for a longitudinal measure with nonindependent groups. According to the pilot study design, which used a small sample and questionnaires on an ordinal scale level, nonparametric paired tests were performed to answer research questions 1 and 2. The repeated-measures mixed ANOVA was performed analyzing research questions 3–4 although equivalent nonparametric tests were decided not suitable. Previous studies have suggested a minimum change of 10–20% for pain on a Numeric Rating Scale, and 30% reduction indicates a clinically relevant difference [57], a level that seems relevant also in this study. The effect size will be interpreted in terms of Cohen's *d* and in the mixed ANOVA in relation to variance. An alpha value was set to a level of 0.05. The statistical analyses of associations were performed using SPSS 23.0 for Windows (IBM Corporation).

### 2.5. Ethical Considerations

This study was carried out in accordance with the World Medical Association-Declaration of Helsinki [58] and the Swedish National Board of Health and Welfare. The study was approved by the Regional Ethical Review Board in Gothenburg (814–13). The participants were informed, both orally and in writing, and were asked to give their informed consent to participate in the study. The participants were also informed that they could interrupt their participation in the study at any time without explanation or consequences. All participants were informed about their ability to contact their home health care providers for guidance if needed.

## 3. Results

Eighteen older adults completed a total of 150 STRENGTH sessions, 5 to 15 (Mdn = 7) each during the intervention period; 12 older adults constituted the control group. There were no statistical differences in gender, mean age, or reported pain levels (see Table 2) or GDS-20 and SOC-13 between the intervention and control group at baseline (T1).

### 3.1. Reported Level of Pain and Wellbeing before and Immediately after a Single STRENGTH

The pain level was reported to be lower (Mdn = 4) after intervention with STRENGTH compared to the time before the intervention (Mdn = 6) on a 0–10 NRS in the BPI-SF (*z* = −6.35, N-Ties = 87, *p* < 0.001). The level of wellbeing was reported higher (Mdn = 8) after intervention with STRENGTH compared to the time before the intervention (Mdn = 7) on a 0–10 NRS (*z* = −5.61, N-Ties = 67, *p* < 0.001). The calculated effect size of decreased pain (*d* = −0.51) and wellbeing level (*d* = −46) represents a medium change, according to Cohen's benchmark criteria.

### 3.2. Longitudinal Effects on Intervention with STRENGTH and Controls

**Figure 2:**
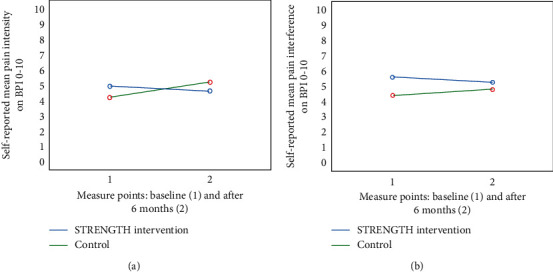
(a) Reported level of pain intensity at baseline and after intervention: STRENGTH (BPI 0–10) (*n* = 18) versus control (*n* = 12). (b) Reported level of pain interference at baseline and after intervention: STRENGTH (BPI 0–10) (*n* = 18) versus control (*n* = 12).

No longitudinal differences could be found for the primary outcome level of pain intensity (F (1.27) = 0.578, *p*=0.454, partial eta^2^ = 0.02) or pain interference (F (1.27) = 0.005, *p* < 0.945, partial eta^2^< 0.01) (see Figures 2(a) and 2(b)). There was an interaction trend on lower pain intensity between the STRENGTH intervention group and the control group over time (F (1.27) = 4.269, *p*=0.049, partial eta^2^ = 0.14) (see Figure 2(a)).

**Figure 3:**
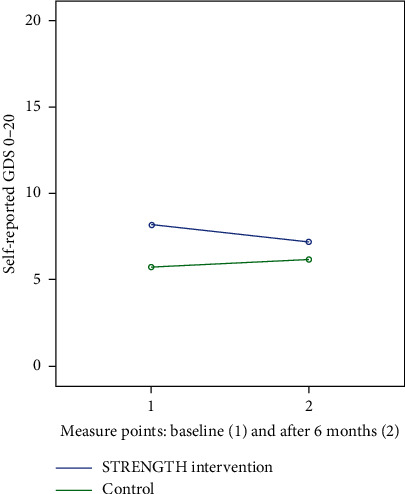
Reported level of depression (GDS) at baseline and after intervention: STRENGTH (*n* = 18) versus control (*n* = 12).

**Figure 4:**
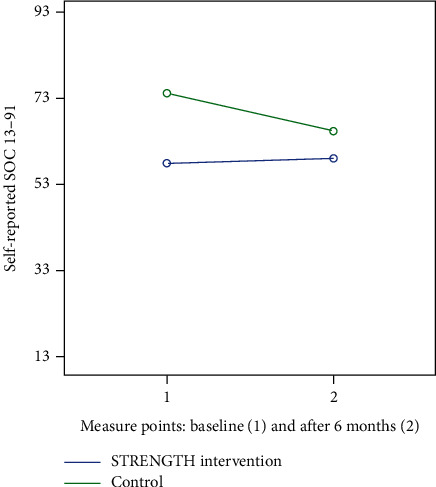
Reported level of sense of coherence (SOC-13) at baseline and after intervention: STRENGTH (*n* = 18) versus control (*n* = 12).

There was no effect on the occurrence or level of depression (according to the GDS-20 instrument) between baseline (T1) and after the six-month study period (T2) with continuous STRENGTH within the intervention group or compared to the control group (F (1.27) = 0.593, *p*=0.448, partial eta^2^ = 0.02). No statistically significant interaction was found on the GDS-20 (F (1.27) = 3.5, *p*=0.072, partial eta^2^ = 0.12) after STRENGTH (see Figure 3). There was no significant difference in the levels of SOC (F (1.27) = 2.888, *p*=0.101, partial eta^2^ = 0.01) but a significant interaction within subjects (F (1.27) 4.803, *p*=0.037, partial eta^2^ = 0.15) (Figure 4) was found. In the STRENGTH intervention group with 18 participants, a power for the primary outcome, pain, turned out to be 0.64 and with an alpha value of 0.05.

## 4. Discussion

STRENGTH has a pain-alleviating effect, according to self-reports, with lower pain levels after an intervention. The single intervention with STRENGTH resulted in a medium change in levels of decreased pain and a medium change (Cohen's criteria) in levels of increased wellbeing in older adults. Also, the level of wellbeing increased after the STRENGTH intervention, according to older adults' self-reports.According to an earlier recommendation, a 10%–20% pain reduction in the baseline NRS score reflects a minimal important change and a reduction of 30% or more represents a clinically important difference [59]. In this study, the pain level went from the corresponding NRS 6 to NRS 4 and was found to be at a reasonable effect. Furthermore, a change of 13 to 18 mm for pain on a Visual Analogue Scale 0–100 mm (comparable to the NRS 0–10) indicates a clinically relevant difference, which should prompt a change in treatment [57], i.e., STRENGTH management for pain alleviation.

From the longitudinal perspective, SOC increased in the intervention group, and no other differences could be shown for intervention with repeated STRENGTHs. The small sample and sometimes lack of continuity might be one potential reason for this result. The tendency of a raising score in the level of depression in the control group compared to the intervention group was a sense experienced by the researchers when collecting data. The repeated measures of pain might also have been influenced by some difficulties that the older adults expressed in scoring both the level of pain and wellbeing before and after the dialogues. However, the older adults expressed in the qualitative interviews [50] that the dialogues helped to ease the pain for some time after the dialogue and thinking back on the dialogue evoked the same feeling. One way to address this and provide continuity on a daily basis is to educate all health care providers to use the approach used in STRENGTH. The control group illuminates the natural deterioration of health problem, which otherwise is a confounding factor when studying older adults longitudinally. This deterioration according to the increased level of SOC and decreased pain for the intervention group seems slower in the intervention group compared to the control group and the interaction lines (Figures 2(a) and 4), which may be interpreted as an effect of STRENGTH, although this accounted for only a small proportion of the variance.

Regarding the pain problem per se, research shows that ways of communication are central in receiving information from older adults, e.g., pain intensity [60]. Questions must be asked repeatedly to understand older adults' descriptions of pain. The influence of the dialogues on the level of pain might have been affected by how older adults live with problems as pain. Earlier studies [32, 33, 50] show that the pain often becomes integral to life itself for the older adults and they view it as a natural part in the process of aging and feel that health care providers and others do as well. Additionally, they do not want to complain or burden others which also adds to their hesitance to talk about pain, all of which might have added to suffering in silence. STRENGTH is a tool used to learn to know the older adult in their situation as a whole which facilitates tailoring and provision of holistic care. An instrument with dimensions, such as functional interference, current pain treatment, treatment effects, and side effects, could be one way to deepen the discussion and knowledge about pain and overall wellbeing in the older adult's situation. However, achieving information about pain can be difficult since older adults, health care professionals, and relatives tend to view pain as a natural part of the aging process, and older adults tend to be stoic and do not want to complain [61, 62]. An effect related to distraction from pain during the STRENGTH intervention could also explain parts of the pain as a reducing effect between the pre- and postmeasures. Also, research shows that older adults focus on living life despite pain but not on pain per se [32]. However, instruments with measures that create a fuller picture of the older adults' situations and wellbeing should be added to the main study with instruments that measure QOL and functional mobility. Furthermore, the educational program for the health care providers of STRENGTH and direct practice seems to be an important factor for success. In addition, STRENGTH is suitable in an interprofessional context, developed, and pilot-tested with different professionals, e.g., nurses, physiotherapists, and occupational therapists. A previous population-based report of self-rated health among older adults living at home concluded that participants aged 65–79 rate their general health as good, but the rate decreases in those over the age of 80 [63]. Thus, intervention with STRENGTH for older adults living with pain should be initiated as early as possible. Today, a commonly used model for dialogues with patients is the Motivational Interviewing (MI) that, in contrast to the holistic approach in STRENGTH, focuses mainly on behavioral changes regarding specific matters, such as quitting smoking or changing lifestyle. Östlund and colleagues studied MI in primary health care and concluded that if the MI was not performed in the right conditions, the dialogue may become only an advisory talk that lacks complex reflection and thus may result in no behavioral change [52]. Therefore, the education program, including reflection and continuous support during STRENGTH for health care providers, is key in obtaining positive effects and changes for the older adults living with pain. Living with long-term pain is not only a physical challenge but also a largely existential manner in which the STRENGTH method is adapted to.

Limitations of the study are the low number and nonrandomized groups of participants. However, this pilot study points out the need for comprehensive study design and provides a basis for feasibility and acceptability of the intervention and instruments used, as well as a power calculation regarding the effect. Also, the one point difference in baseline pain level may be noted as a bias even with no significant difference between the study groups. There was no effort to control for gender in this study. Long-term musculoskeletal pain is known to be more prevalent among women, and women live longer than most men [64, 65], which led to a predominance of women among the participants, an outcome that could be viewed as a limitation. The validity was further enhanced since, if necessary, time was given to participants to explain questions regarding the instruments and the ability to reflect upon adding anything before the questionnaire was collected. The findings, however, may have been influenced by the older adults' tendency to be stoic [61, 62] with a decreased report of pain levels.

A strength in this pilot study is that all participants despite impairments were able to respond to the questionnaires. The researchers read the questions in the questionnaires and helped the older adults to write their responses if needed which is importantly viewed from the perspectives of equal rights and ageism. Another strength of the design is that the participants are their own control with a repeated measure and also a control group to evaluate the intervention over time. The control group was also recruited from a community with staff who were not involved in the STRENGTH intervention. The individual marks the appropriate point on the scale [66]. The instrument is suitable for assessing pain [59, 67, 68] and most individuals have no difficulty using it [67, 69]. SOC seems to have a main moderating or mediating role in the explanation of wellbeing as a contributor to the development and maintenance of people's health but may not explain the overall health alone [70].

## 5. Conclusions

This pilot study supports the pain-alleviating effect and increased wellbeing of reflective STRENGTH-giving dialogues in older adults. This result is in congruence with the interviews from an earlier study qualitatively evaluating STRENGTH. No longitudinal effect differences on the primary outcome variable pain were found. Furthermore, the study provides a basis for future power calculation in relation to the instruments used measuring symptoms present during long-term pain. The focus in this study is older adults with long-term pain but the method may have the potential to be implemented as a useful intervention in the provision of health care and social services to older adults. However, further research is needed to investigate the transferability of the method STRENGTH to other contexts about age groups, health problems, settings, and professions.

## Figures and Tables

**Table 1:** Key dimensions of the reflective STRENGTH-giving dialogue.

Reflective STRENGTH-giving dialogue
S—State the current situation
T—Transition from “one to I” and take charge in the situation
R—Reflect upon possibilities and choices
EN—Engagement in fulfilling small and large life projects that give joy and meaning in life
G—Get inner strength and courage
T—Tactful and challenging approach
H—Holistic perspective

**Table 2:** Background characteristics and baseline pain level for the reflective STRENGTH-giving dialogue intervention group and the control group.

	Intervention group (STRENGTH) (*n* = 18)	Control group (*n* = 12)
	*n*	*n*
*Gender*		
Female	13	8
Male	5	4
*Age (yr) Md*		
74	3	3
75–84	9	4
85+	6	5
*Marital status*		
Married	3	2
Widowed	11	10
Divorced	2	
Single	2	
*Living situation*		
Single	15	10
Cohabiting	3	2
*Baseline self-reported level of pain latest 24 h (BPI-SF* ^*a*^)		
Worse pain Md	8	7
Least pain Md	3	2
Average pain Md	5	4

^a^Pain intensity was measured with the Brief Pain Inventory-Short Form 0 to 10 scale with 0, no pain, and 10, pain as bad as you can imagine.

## Data Availability

The datasets generated and analyzed during the current study are not publicly available due to legislation in community-based care but are available from the corresponding author upon reasonable request.
